# High occurrence of *Enterococcus faecalis*, *Enterococcus faecium*, and *Vagococcus lutrae* harbouring oxazolidinone resistance genes in raw meat-based diets for companion animals – a public health issue, Switzerland, September 2018 to May 2020

**DOI:** 10.2807/1560-7917.ES.2023.28.6.2200496

**Published:** 2023-02-09

**Authors:** Magdalena Nüesch-Inderbinen, Lore Heyvaert, Andrea Treier, Katrin Zurfluh, Nicole Cernela, Michael Biggel, Roger Stephan

**Affiliations:** 1 Institute for Food Safety and Hygiene, Vetsuisse Faculty, University of Zurich, Zurich, Switzerland; 2 Department Veterinary and Biosciences, Faculty Veterinary Medicine, University of Ghent, Ghent, Belgium

**Keywords:** raw pet food, *Enterococcus*, *Vagococcus*, linezolid resistance, dogs, dog owners, public health

## Abstract

**Introduction:**

Enterococci harbouring genes encoding resistance to florfenicol and the oxazolidinone antimicrobial linezolid have emerged among food-producing animals and meat thereof, but few studies have analysed their occurrence in raw meat-based diets (RMBDs) for pets.

**Aim:**

We aimed to examine how far RMBDs may represent a source of bacteria with oxazolidinone resistance genes.

**Methods:**

Fifty-nine samples of different types of RMBDs from 10 suppliers (three based in Germany, seven in Switzerland) were screened for florfenicol-resistant Gram-positive bacteria using a selective culture medium. Isolates were phenotypically and genotypically characterised.

**Results:**

A total of 27 *Enterococcus faecalis*, *Enterococcus faecium*, and *Vagococcus lutrae* isolates were obtained from 24 of the 59 samples. The *optrA*, *poxtA*, and *cfr* genes were identified in 24/27, 6/27 and 5/27 isolates, respectively. Chloramphenicol and linezolid minimum inhibitory concentrations (MICs) ranged from 24.0 mg/L–256.0 mg/L, and 1.5 mg/L–8.0 mg/L, respectively. According to the Clinical and Laboratory Standards Institute (CLSI) breakpoints, 26 of 27 isolates were resistant to chloramphenicol (MICs ≥ 32 mg/L), and two were resistant to linezolid (MICs ≥ 8 mg/L). Multilocus sequence typing analysis of the 17 *E. faecalis* isolates identified 10 different sequence types (ST)s, with ST593 (n = 4 isolates) and ST207 (n = 2 isolates) occurring more than once, and two novel STs (n = 2 isolates). *E. faecium* isolates belonged to four different STs (168, 264, 822, and 1846).

**Conclusion:**

The high occurrence in our sample of Gram-positive bacteria harbouring genes encoding resistance to the critical antimicrobial linezolid is of concern since such bacteria may spread from companion animals to humans upon close contact between pets and their owners.

**Key public health message**

**What did you want to address in this study?**

Feeding pets raw meat-based diets (RMBDs) is a growing trend among pet owners. Unfortunately, these diets may be contaminated with parasites, pathogenic bacteria, and antibiotic resistant bacteria, including bacteria that carry genes for resistance to linezolid, a last-resort drug to treat severe infections in people. Our aim was to examine to if RMBDs represent a possible source of bacteria with linezolid resistance genes.

**What have we learnt from this study?**

In 24 of 59 RMBD samples mostly originating from Switzerland and Germany, that we examined, we identified bacteria with genes which may confer resistance to linezolid. Some of these bacteria were closely related to bacteria that cause infections in humans worldwide.

**What are the implications of your findings for public health?**

The occurrence of bacteria harbouring linezolid resistance genes in RMBDs in our sample may pose a possible risk to human health. These bacteria could spread from companion animals to their owners or to any other individual in close contact with the companion animals. Veterinary and public-health agencies, RMBD suppliers, and pet owners should be aware of this.

## Introduction

Feeding companion animals raw meat has become well established among many cat and dog owners who wish to provide their pets with a natural and healthy alternative to conventional pet food [1,2]. Raw meat-based diets (RMBDs), also known as Biologically Appropriate Raw Food (BARF), usually contain meat by-products originating from food-producing animals slaughtered for human consumption including muscle and organ meats, and meaty bones. Since RMBDs are not cooked or pasteurised, concerns have been raised that pathogens occurring in RMBDs pose a risk of infectious disease to humans and their pets [3]. RMBDs have also been identified as a potential source of antimicrobial resistant bacteria [1]. Thus, although not destined for human consumption, handling of RMBDs and the close contact and complex interactions of pets with their owners and their environment represent possibilities of transmission of resistant bacteria from raw pet food products to humans. This is of particular relevance, since antimicrobial resistance (AMR) is currently one of the most important threats to human and animal health worldwide, affecting people, animals, and the environment [4].

Because of the use and overuse of antimicrobial agents in domestic livestock production, food-producing animals have emerged as an important reservoir for AMR [5]. In particular, the extensive use of florfenicol, a fluorinated phenicol derivative of chloramphenicol, has selected florfenicol-resistant bacteria including *Enterococcus* and *Staphylococcus* spp. that carry genes associated with florfenicol resistance such as *fexA* and *fexB* (phenicol exporter genes), *optrA* and *poxtA* (genes encoding ribosomal protection proteins), and *cfr* (a 23SrRNA methyltransferase gene) [6]. Notably, the horizontally transferrable genes *optrA*, *poxtA* and *cfr* provide resistance not only to phenicols but also to oxazolidinones, resulting in cross-resistance to linezolid [7,8]. Linezolid is an oxazolidinone antimicrobial that was first introduced into therapy in 2000 and which is currently considered a last resort drug for treating severe infections caused by Gram-positive pathogens such as vancomycin-resistant enterococci (VRE), meticillin-resistant *Staphylococcus aureus* (MRSA) and multidrug-resistant pneumococci [9]. Linezolid is also considered by the World Health Organization (WHO) to be a critically important antimicrobial for use in human medicine [10].

Increasing reports on linezolid resistant enterococci detected in food-producing animals and meat thereof [9,11,12], together with the growing popularity of feeding pets RMBDs, prompted us to assess the occurrence of florfenicol resistant bacteria among commercially available raw pet food in Switzerland.

## Methods

### Sampling and screening for florfenicol-resistant bacteria

A convenience sample of 59 different types of RMBDs was commercially purchased by the investigators in six locations within a radius of 300 km of the laboratory in Switzerland, or via Internet shops during September 2018 and May 2020. The products originated from 10 different suppliers, designated A–J. Suppliers A (10 samples), G (10 samples), and I (four samples) were pet shop stores located in Germany. Suppliers B (eight samples), C (six samples), F (eight samples), H (seven samples), and J (one sample) were pet shops stores based in Switzerland. Supplier D (four samples) was a one-person Swiss RMBD producing business which was officially certified based on hazard analysis and critical control points (HACCP) hygiene standards by the county veterinary office. Supplier E (one sample) was a butcher who slaughters and sells regionally farmed Swiss meat and processes the slaughter by-products to in-house produced RMBDs. All products were purchased frozen or shipped frozen to the laboratory and stored until analysis. The tested products contained either pure muscle or pure organ meat, mixed muscle and organ meat products, or meat supplemented with plant ingredients. Only samples that contained uncooked meat or organs that had not undergone any treatment, such as pasteurisation or drying, were purchased. Only RMBDs intended for dogs were included.

Samples were categorised as beef (n = 17), poultry (n = 15), horse (n = 8), lamb (n = 6), fish (n = 5), rabbit (n = 4), and venison (n = 4). The suppliers' source declarations indicated that overall, the majority (54/59) of the RMBDs were produced using meat sourced from European countries or from Australia. For one of the eight horse meat samples and for four of the five fish samples (salmon) the countries of origin remained unknown. One fish RMBD sample contained perch and pangasius from Estonia and Vietnam.

A subset of 10 g of each sample was homogenised at a 1:10 ratio in brain heart infusion (BHI) broth with 6.5% NaCl (Oxoid, Pratteln, Switzerland) and incubated for 18–24 hours at 37 °C. One loopful of the enriched culture was streaked on Bile Aesculin Azide (BAA) agar (Merck, Darmstadt, Germany), with 10 mg/L florfenicol (Sigma-Aldrich, Buchs, Switzerland) and incubated under aerobic conditions for 48 hours at 37 °C. All colonies with morphological characteristics similar to *Enterococcus* spp. (small transparent colonies with brown-black halos) were subcultured on BAA agar with 10 mg/L florfenicol for 48 hours at 37 °C and single colonies subcultured on plate count agar (Difco, Becton Dickinson, Allschwil, Switzerland) for 24 hours at 37 °C. These isolates were considered florfenicol resistant and stored in 20% glycerol at − 20 °C for further analysis.

### Identification of *Enterococcus* and *Vagococcus* species and screening for oxazolidinone resistance genes

Species identification was performed by matrix-assisted laser desorption–ionisation time of flight mass spectrometry (MALDI-TOF-MS, Bruker Daltonics, Billerica, Massachusetts, United States (US)) using Compass FlexControl version 3.4 software with the Compass database version 4.1.80. The presence of *optrA*, *poxtA,* and *cfr* was established by singleplex PCR using custom synthesised primers (Microsynth, Balgach, Switzerland), and conditions described previously [13].

### Antimicrobial susceptibility testing

Minimal inhibitory concentrations (MICs) of chloramphenicol and linezolid were determined using Etest (bioMérieux, Marcy l’Etoile, France). Results were interpreted using the 2022 Clinical and Laboratory Standards Institute (CLSI) enterococci susceptibility breakpoints for broth microdilution, with resistance breakpoints set at ≥ 32 mg/L for chloramphenicol, and ≥ 8 mg/L for linezolid, respectively [14]. *Enterococcus faecium* National Collection of Type Cultures (NCTC, United Kingdom (UK) Health Security Agency) 13923 and *E. faecalis* NCTC 12697 were used as quality control strains.

### Whole genome sequencing and genome analysis

Whole genome sequences were determined using short-read and long-read sequencing. Isolates were grown on sheep blood agar (Difco, Becton Dickinson, Allschwil, Switzerland), and genomic DNA was extracted using the MasterPure complete DNA and RNA Purification Kit (Lucigen Corporations, Wisconsin, US). For short-read sequencing, libraries were prepared using the Nextera DNA Flex Library Preparation Kit (Illumina, San Diego, California, US), and sequencing was performed on the Illumina MiniSeq platform with 2 × 150 bp paired-end chemistries. For long-read sequencing, libraries were prepared using an SQK-LSK109 Ligation Sequencing Kit (Oxford Nanopore Technologies, Oxford, UK) and sequenced on a MinION Mk1B device using the FLO-MIN106 (R9) flow cell (Oxford Nanopore Technologies, Oxford, UK).

Hybrid assemblies were generated with Unicycler v.0.5 using default settings (https://github.com/rrwick/Unicycler). Genes were annotated using Prokka v.1.14.6 (https://github.com/tseemann/prokka) and isolates were typed in silico using PubMLST (https://pubmlst.org/). Antimicrobial resistance genes and plasmid replicons were identified using ABRicate 1.0.1 (https://github.com/tseemann/abricate) in combination with the ResFinder (https://bitbucket.org/genomicepidemiology/resfinder_db/) and PlasmidFinder (https://bitbucket.org/genomicepidemiology/plasmidfinder/) databases, respectively. Plasmid replicon families were determined based on conserved domains identified in homologous replicon sequences identified in the National Center for Biotechnology Information (NCBI) Nucleotide collection using Basic Local Alignment Search Tool (BLAST; http://www.ncbi.nlm.nih.gov/books/NBK21097/).

Reads were also queried for mutations in the 23S rRNA genes associated with linezolid resistance (G2505A and G2576T) using LRE-finder 1.0 (https://cge.food.dtu.dk/services/LRE-finder/).

## Results

### Isolation of florfenicol resistant Gram-positive cocci and screening for oxazolidinone-resistance genes

Overall, florfenicol resistant bacteria were found in 24 of the 59 RMBD samples, including six of the 17 samples containing beef, seven of the 15 poultry-based diets, three of the eight horse meat samples, one of the six lamb meat diets, two of the five fish samples, three of the four rabbit meat samples, and two of the four RMBD samples consisting of venison. Three of the 59 samples (horse meat sample LS05, poultry sample AT40, and rabbit meat sample AT46) yielded two distinct isolates each, resulting in a total of 27 florfenicol resistant isolates. Species identification revealed that the 27 isolates included *E. faecalis* (n = 17), *E. faecium* (n = 4), and *Vagococcus lutrae* (n = 6) (Table 1). Of the samples yielding two distinct isolates, LS05 and AT40 contained two different *E. faecalis* each, and AT46 contained one *E. faecalis* and one *V. lutrae* (Table 1).

**Table 1:** Distribution of *Enterococcus faecalis*, *Enterococcus faecium*, and *Vagococcus lutrae* habouring oxazolidinone resistance genes among samples of commercially available raw meat-based diets (RMBDs) for companion animals Switzerland, September 2018–May 2020 (n = 24)

Isolate ID	Sample ID	Meat category	Country of origin of the raw meat	Supplier	Oxazolidinone resistance gene		
					*cfr*	*optrA*	*poxtA*
*Enterococcus faecalis*							
AT04	AT 04	Beef	Switzerland	F	−	+	−
AT09	AT 09	Poultry	Germany	A	−	+	−
AT22	AT 22	Poultry	Switzerland	C	−	+	−
AT29	AT 29	Lamb	Switzerland	H	−	+	−
AT34	AT 34	Fish	Unknown	H	−	+	−
AT39	AT 39	Horse	Germany	I	+	−	+
AT40a	AT 40	Poultry	Germany	G	−	+	−
AT40b	AT 40	Poultry	Germany	G	+	+	−
AT41	AT 41	Poultry	Germany	G	−	+	−
AT43a	AT 43	Beef	Germany	G	−	+	−
AT46a	AT 46	Rabbit	Germany	G	−	+	−
AT48	AT 48	Poultry	Germany	G	−	+	−
AT49a	AT 49	Venison	Germany	G	+	+	−
AT50	AT 50	Rabbit	Australia/EU/Switzerland	B	−	+	−
LS05–1	LS 05	Horse	Unknown	J	−	+	−
LS05–2	LS 05	Horse	Unknown	J	+	+	+
LS06–1	LS 06	Beef	Switzerland	F	−	+	−
*Enterococcus faecium*							
AT02	AT 02	Beef	Germany	A	+	+	+
AT44	AT 44	Venison	Germany	G	−	−	+
AT45b	AT 45	Poultry	Germany	G	−	−	+
AT47b	AT 47	Fish	Unknown	G	−	+	+
*Vagococcus lutrae*							
AT03	AT 03	Horse	Germany	A	−	+	−
AT15	AT 25	Beef	Switzerland	C	−	+	−
AT19	AT 19	Beef	Australia/EU/Switzerland	B	−	+	−
AT32	AT 32	Rabbit	Switzerland	H	−	+	−
AT33	AT 33	Poultry	Switzerland	H	−	+	−
AT46b	AT 46	Rabbit	Germany	G	−	+	−

EU: European Union; ID: identity.

A plus sign (+) indicates the presence of the gene, a minus sign (−) the absence of the gene, determined by PCR.

RMBDs containing resistant bacteria originated from eight of the 10 suppliers, with suppliers D and E representing the exceptions.

PCR screening demonstrated the presence of one or more oxazolidinone resistance genes in all florfenicol resistant isolates (Table 1). The *optrA* was present in 24/27 of the isolates, consisting of *E. faecalis* (n = 16), *E. faecium* (n = 2), and *V. lutrae* (n = 6). The *poxtA* gene was identified in a total of six of the 27 isolates, including *E. faecalis* (n = 2), and *E. faecium* (n = 4). The *cfr* gene was found in five of the 27 isolates comprising *E. faecalis* (n = 4), and *E. faecium* (n = 1) (Table 1).

### Minimal inhibitory concentrations

All 27 isolates were tested for their susceptibility to chloramphenicol and linezolid (Table 2). The MICs of chloramphenicol and linezolid ranged from 24.0 to > 256.0 mg/L, and 1.5–8.0 mg/L, respectively. Twenty-six of the 27 isolates had chloramphenicol MIC values above the CLSI breakpoint of ≥ 32 mg/L, and two of the 27 isolates were classified as resistant to linezolid according to the CLSI breakpoint (MIC ≥ 8 mg/L) [14]. Both linezolid resistant isolates (isolates AT15 and AT50, respectively) were also resistant to chloramphenicol (Table 2)

**Table 2:** Characteristics of 21 *Enterococcus* spp. and six *Vagococcus lutrae* harbouring oxazolidinone resistance genes and originating from raw meat-based diets (RMBDs) for companion animals, Switzerland, September 2018–May 2020 (n = 27)

Isolate ID	Species	MLST	MIC (mg/L)		Oxazolidinone resistance determinants^b^; location (plasmid replicon type)	Other antimicrobial resistance genes^c^	GenBank accession number
			CL^a^	LZ^a^			
AT39	*E. faecalis*	86	256.0	3.0	*cfr*(D)*;* plasmid pAT39-b (rep1, rep2) *poxtA;* plasmid pAT39-b (rep1, rep2)	*ant(6)-Ia*, *aph(3')-III, cat*, *dfrG*, *erm*(B), erm(B), *fexB*, *lsa*(A), *tet*(L), *tet*(M)	GCA_023299625.1
AT50	*E. faecalis*	256	256.0	8.0	*optrA* [KD]; plasmid pAT50-a (rep9a, rep7a, rep27)	*aph(3')-III*, *cat*, *dfrG*, *erm* (A), *erm* (B), *fexA*, *lsa* (A), *str*, *tet* (L), *tet* (M)	GCA_023299645.1
AT40a	*E. faecalis*	476	256.0	4.0	*optrA* [none]; chromosome	*aac(6')-aph(2”)*, *ant(6)-Ia*, *ant(9)-Ia*, *aph(3')-II*I, *cat*, *erm*(A), *erm*(B), *fexA, lnu*(B), *lsa*(A), *lsa*(E), *tet*(L), *tet(*M)	GCA_023299585.1
AT41	*E. faecalis*	631	256.0	4.0	*optrA* [none]; chromosome	*aac(6')-aph(2”)*, *ant(9)-Ia*, *aph(3')-III*, *cat*, *dfrG*, *erm*(A), *erm*(B), *fexA*, *lsa*(A), *tet*(L), *tet*(M)	GCA_023300025.1
AT48	*E. faecalis*	474	256.0	3.0	*optrA* [EDD]; chromosome	*aac(6')-aph(2”)*, *ant(6)-Ia*, *ant(9)-Ia*, *aph(3')-III*, *cat*, *dfrG*, *erm*(A), *erm*(B), *fexA*, *lnu*(B), *lsa*(A), *lsa*(E), *tet*(L), *tet*(M)	GCA_023299425.1
LS06–1	*E. faecalis*	369	256.0	4.0	*optrA* [EDD]; chromosome	*ant(9)-Ia*, *aph(3')-III*, *cat*, *dfrG*, *erm*(A), *erm*(B), *fexA*, *lsa*(A), *tet*(L), *tet*(M)	GCA_023299525.1
AT34	*E. faecalis*	376	256.0	6.0	*optrA* [RDK]; plasmid pAT34-a (rep9a)	*cat, erm* (B), *fexA*, *lsa*(A), *tet* (L), *tet* (M)	GCA_023299705.1
LS05–1	*E. faecalis*	1263	256.0	6.0	*optrA* [RDK]; plasmid pLS05-1-a (rep9a, repUS43)	*cat*, *erm* (B), *fexA*, *lsa*(A), *tet* (L), *tet* (M)	GCA_023299465.1
AT09	*E. faecalis*	593	256.0	1.5	*optrA* [EYDNDM]; chromosome	*ant(6)-Ia*, *aph(3')-III, cat*, *dfrG*, *erm*(B), *fexA*, *lsa*(A), *str*, *tet*(L), *tet*(M)	GCA_023299545.1
AT29	*E. faecalis*	593	256.0	1.5	*optrA* [EYDNDM]; chromosome	*cat*, *dfrG*, *erm*(B), *fexA*, *lsa*(A), *str*, *tet*(L), *tet*(M)	GCA_023299665.1
AT46a	*E. faecalis*	593	256.0	2.0	*optrA* [EYDNDM]; chromosome	*ant(6)-Ia*, *aph(3')-III*, *cat*, *dfrG*, *erm*(B), *fexA*, *lsa*(A), *str*, *tet*(L), *tet*(M)	GCA_023299605.1
AT49a	*E. faecalis*	593	256.0	2.0	*optrA* [EYDNDM]; chromosome *cfr*(D); plasmid pAT49a-a (rep9a, repUS43, rep7a)	*cat*, *erm* (B), *fexA*, *lsa*(A), *str*, *tet* (L), *tet* (M)	GCA_023299225.1
AT40b	*E. faecalis*	16	192.0	3.0	*optrA* [EYKKCDVASKELYNKQLEIG]; plasmid pAT40b-b (rep9c); *cfr*(D); plasmid pAT40b-c (rep9a)	*aac(6')-aph(2”)*, *ant(6)-Ia, aph(3')-III*, *dfrG*, *erm*(B), *fexA, lnu(*B), *lsa*(A), *lsa*(E), *tet*(M)	GCA_023299505.1
LS05–2	*E. faecalis*	1008	256.0	4.0	*optrA* [EYNKWKVDASKELYNKQLEIG]; chromosome *poxtA*; chromosome *cfr;* chromosome	*ant(9)-Ia*, *fexB*, *lsa*(A)	GCA_023299485.1
AT22	*E. faecalis*	1262	32.0	2.0	*optrA* [EDD_2]; chromosome	*fexB, lnu*(G), *lsa*(A), *tet*(L), *tet*(M), *tet*(O/W/32/O)	GCA_023299685.1
AT04	*E. faecalis*	207	256.0	1.5	*optrA* [EDD_2]; chromosome	*ant(6)-Ia*, *aph(3')-III*, *erm*(B), *fexB*, *lsa(*A), *tet*(L), *tet*(M), *tet*(O/W/32/O)	GCA_023300165.1
AT43a	*E. faecalis*	207	96.0	3.0	*optrA* [EDD_2]; chromosome	*fexB*, *lsa*(A), *tet(*L), *tet*(M), *tet*(O/W/32/O)	GCA_023299785.1
AT47b	*E. faecium*	822	192.0	3.0	*optrA* [DD_3]; chromosome *optrA* [DD_3]; plasmid pAT47b-a (repUS15, rep1) *poxtA;* plasmid pAT47b-b (rep2)	*aac(6')-Ii*, *ant(6)-Ia*, *clpL*, *dfrG*, *erm* (A), *fexB*, *lnu* (B), *lsa* (E), *msr*(C), *tet* (L), *tet* (M)	GCA_023299325.1
AT02	*E. faecium*	1846	256.0	6.0	*optrA* [DDM]; plasmid pAT02-a (n.i.) *poxtA;* plasmid pAT02-b (rep2, rep29, repUS43) *cfr*(D); plasmid pAT02-c (rep1)	*aac(6')-Ii, clpL*, *erm* (B), *fexA*, *fexB*, *lnu*(G), *msr*(C)	GCA_023299805.1
AT45b	*E. faecium*	168	48.0	3.0	*poxtA;* plasmid pAT45b-b (rep29)	*aac(6')-Ii*, *ant(6)-Ia*, *aph(3')-III*, *cat*(pC233), *clpL*, *erm*(B), *fexB*, *msr*(C), *tet*(L), *tet*(M)	GCA_023299725.1
AT44	*E. faecium*	264	32.0	2.0	*poxtA;* plasmid pAT44-b (rep2)	*aac(6')-aph(2”)*, *aac(6')-Ii*, *ant(6)-Ia*, *aph(3')-III*, *clpL*, *dfrG*, *erm* (B), *fexB*, *lnu*(B), *lnu(*G), *lsa*(E), *msr*(C), *tet* (L), *tet* (M)	GCA_023299745.1
AT19	*V. lutrae*	NA	> 256.0	3.0	*optrA* [none]*;* chromosome	*aac(6')-aph(2”)*, *aac(6')-aph(2”)*, *ant(6)-Ia*, *aph(3')-III, cat*(pC221), *dfrG*, *erm*(B), *fexA*, *lnu(*B), *lsa*(E), *tet*(L), *tet*(M)	GCA_023299345.1
AT32	*V. lutrae*	NA	24.0	3.0	*optrA* [none]*;* chromosome	*aac(6')-aph(2”)*, *ant(6)-Ia*, *aph(3')-III*, *cat*(pC221), *erm*(B), *fexA*, *tet*(M)	GCA_023299765.1
AT33	*V. lutrae*	NA	64.0	4.0	*optrA* [none]*;* chromosome	*aac(6')-aph(2”)*, *ant(6)-Ia*, *aph*(3')-III, *cat*(pC221), *erm*(B), *fexA*, *tet*(M)	GCA_023299445.1
AT15	*V. lutrae*	NA	> 256.0	8.0	*optrA* [EDM]; plasmid pAT15-a (n.i.)	*aac(6')-aph(2”)*, *ant(6)-Ia*, *aph(3')-III*, *cat*(pC221), *erm*(B), *fexA*, *tet*(M)	GCA_023299565.1
AT46b	*V. lutrae*	NA	> 256.0	4.0	*optrA* [EDM]; plasmid pAT46b-a (n.i.)	*aac(6')-aph(2”)*, *ant(6)-Ia*, *fexA*, *lnu*(B), *lsa*(E), *tet*(M)	GCA_023299185.1
AT03	*V. lutrae*	NA	> 256.0	2.0	*optrA* [EYDNDM]*;* chromosome	*aac(6')-aph(2”)*, *ant(6)-Ia*, *dfrG*, *erm*(B), *fexA*, *lnu*(B), *lsa*(E), *mef*(A), *msr*(D), *tet*(M)	GCA_023300045.1

CL: chloramphenicol; ID: identity; LZ: linezolid, MIC: minimal inhibitory concentration; MLST: multilocus sequence type; NA: not applicable; n.i.: not identified.

^a^ Breakpoints for resistance were ≥ 32 mg/L for CL and ≥ 8mg/L for LZ, according to Clinical and Laboratory Standards Institute (CLSI) [14].

^b^ The *optrA* gene is identical to wild-type *optrA*_E349_ (GenBank accession number: KP399637) [8]. Amino-acid changes corresponding to *optrA* gene mutations are indicated in square brackets for each OptrA variant, corresponding to the nomenclature of Schwarz et al. [9]. Amino-acid substitutions and their positions are detailed in Table 3.

^c^ Antimicrobial resistance genes co-located on plasmids harbouring oxazolidinone resistance genes are underlined.

### Identification of *optrA, poxtA,* and *cfr* variants

For the *optrA*-harbouring isolates, nt sequences were compared with the wild-type *optrA*_E349_ (GenBank accession number: KP399637) [8], and OptrA variants were defined based on alterations in the deduced amino-acid sequences according to Schwarz et al. [9]. A total of 11 OptrA variants (including the wild type) were identified among the 24 *optrA*-positive isolates (Table 2). The *optrA* genes were found to be carried either by chromosomal or plasmid DNA (Table 2). Among the 21 enterococci, the OptrA EYDNDM variant was the most common (n = 4), followed by OptrA EDD_2 (n = 3). Among the six *V. lutrae*, the wild-type OptrA variant was the most prevalent (n = 3). The OptrA variants, their amino-acid substitutions and the distribution among the isolates described in this study are listed in Table 3.

**Table 3:** OptrA variants detected in 16 *Enterococcus faecalis*, two *Enterococcus faecium*, and six *Vagococcus lutrae* isolated from RMBD samples, Switzerland, September 2018–May 2020 (n = 11 variants)

OptrA variant	Amino-acid substitution(s) and positions^a^	Species (number of isolates)	Reference(s) to where the variants are described
Wild type^b^	None	*E. faecalis* (2), *V. lutrae* (3)	[8,35]
DD_3	Y176**D**, G393**D**	*E. faecium (1)*	[9]
DDM	Y176**D** G393**D,** I622**M**	*E. faecium (1)*	[9]
EDD	K3**E**, Y176**D**, G393**D**	*E. faecalis (2)*	[34]
EDD_2	K3**E**, G40**D**, G393**D**	*E. faecalis (3)*	This study
EDM	K3**E**, Y176**D**, I622**M**	*V. lutrae (2)*	[34]
EYDNDM	K3**E**, N12Y, Y176**D**, D247**N**, G393**D**, I622**M**	*E. faecalis (4), V. lutrae (1)*	[9]
EYKKCDVASKELYNKQLEIG	K3**E**, N12**Y**, E37**K**, N122**K**, Y135**C**, Y176**D**, A35**0V**, V395**A**, A396**S**, Q509**K**, Q541**E**, M552**L**, N560**Y**, K562**N**, Q565**K**, E614**Q**, I627**L**, D633**E**, N640**I**, R650**G**	*E. faecalis (1)*	[9]
EYNKWKVDASKELYNKQLEIG	K3**E**, N12**Y**, G40**N**, N122**K**, Y135**W**, I287**K**, A350**V**, G393**D**, V395**A**, A396**S**, Q509**K,** Q541**E,** M552**L**, N560**Y**, K562**N**, Q565**K**, S614**Q**, I627**L**, D633**E**, N640**I,** R659**G**	*E. faecalis (1)*	This study
KD	T112**K**, Y176**D**	*E. faecalis (1)*	[9]
RDK	I104**R**, Y176**D**, E256**K**	*E. faecalis (2)*	[9]

^a^ Substituted amino acids are shown in bold.

^b^ Nt sequences correspond to *optrA*_E349_ (GenBank accession number: KP399637) and *optrA_5* [8].

Among the six *poxtA*-positive isolates, whole genome sequencing analysis identified the *poxtA* gene corresponding to that from *S. aureus* AOUC-0915 (GenBank accession number: MF095097) [7], either encoded on the chromosomes or carried by plasmids (Table 2).

Among the five *cfr*-positive isolates, one *E. faecalis* contained a chromosomal *cfr* corresponding to that encoded on plasmid pSCFS3 from *S. aureus* (GenBank accession number: AM086211.1) [15]. The remaining isolates carried *cfr*(D) (GenBank accession number: NG_067192) [16] located on plasmids (Table 2).

### Detection of other antimicrobial resistance genes

The phenicol resistance gene *fexA* was identified in 19 isolates, including *E. faecalis* (n = 12), *E. faecium* (n = 1) and *V. lutrae* (n = 6) (Table 2). The *fexB* gene was found in nine isolates, including *E. faecalis* (n = 5), *E. faecium* (n = 4) (Table 2 *)*. Other antimicrobial resistance genes conferring resistance to phenicols, lincosamides, macrolides, streptogramins, tetracycline, and diaminopyrimidines are listed in Table 2.

None of the isolates in this study revealed the presence of mutations in domain V of the 23S rRNA gene which are associated with linezolid resistance [9].

### Molecular typing of *Enterococcus faecalis* and *Enterococcus faecium*

MLST analysis for the 17 *E. faecalis* identified13 different STs, with ST593 (n = 4) and ST207 (n = 2) occurring more than once (Table 2). Interestingly, all four *E. faecalis* ST593, isolated from RMBDs from various types of meat, carried the OptrA EYDNDM variant, while both *E. faecalis* ST207 isolated from beef RMBDs carried OptrA EDD_2. Two novel STs were identified among the *E. faecalis*, including ST1262 and ST1263 (Table 2).

The four *E. faecium* isolates were assigned to four different STs (ST168, ST264, ST822, and ST1846 respectively, Table 2).

## Discussion

The present study includes an assessment of the occurrence of florfenicol resistant bacteria among 59 RMBDs consisting of various types of meat obtained from different suppliers in two countries. A total of 27 isolates, all harbouring oxazolidinone resistance genes, were retrieved and analysed, including 17 *E. faecalis*, four *E. faecium*, and six *V. lutrae.* To the best of our knowledge, this is the first report of *optrA*-harbouring *V. lutrae* isolated from raw pet food, although *cfr*(D) + *optrA*-carrying *V. lutrae* has recently been isolated from a diseased pig in China [17]. Moreover, plasmid-mediated transfer of *cfr* from porcine *V. lutrae* to *E. faecalis* has been documented [17]. Therefore, the occurrence of *V. lutrae* harbouring *optrA* in multiple RMBDs consisting of various meat types (beef, horse, poultry, or rabbit meat respectively) suggests that this species, although rarely pathogenic to humans, may play a role in the dissemination of oxazolidinone resistance genes to clinically important pathogens.

The overall prevalence of florfenicol resistant isolates among the RMBD samples in this study was 24/59. Regarding enterococci, the proportion of RMBDs containing *cfr*-, *optrA*- or *poxtA*-harbouring isolates in this study was 19/59, which is lower than the 64% reported in a recent study focused on multidrug-resistant enterococci in dog food in Portugal [18]. Although differences in study design and testing methodologies must be considered when comparing these results, our data support the previous finding that *cfr*- *optrA*- or *poxtA*-harbouring enterococci are highly prevalent among RMBDs. Conversely, while recent years have seen an increase in popularity of feeding pets raw meat, many owners underestimate the risks posed by the feeding of RMBDs for both animal and human health [2], highlighting the need to promote awareness among pet owners, veterinary and public-health agencies, and RMBD suppliers to mitigate the emergence and dissemination of linezolid-resistant enterococci in the future. Moreover, the high occurrence of florfenicol resistant isolates in raw pet food made from meat primarily of European origin is concerning and points to the importance of rational use of florfenicol in the agricultural sector in Europe and worldwide to mitigate co-selection of clinically relevant oxazolidinone resistance genes in different bacterial species.

Various *E. faecalis* and *E. faecium* STs identified in this study have been found in clinical, animal, and environmental samples worldwide [8,11,19-28]. For example, *optrA*-carrying *E. faecalis* ST16 has been recovered from human clinical samples in China, Greece, and Denmark [19-22], from food-producing animals [29], and from the aquatic environment [30]. Specifically, *E. faecalis* ST16 (isolate ID 40b) harbouring the OptrA EYKKCDVASKELYNKQLEIG variant in this study was identified previously in a human clinical isolate in the UK [31]. The detection of *optrA*-harbouring *E. faecalis* ST16 in RMBDs provides further evidence of its distribution across different sectors including humans, food-producing animals, meat, and the environment.

Likewise, *E. faecalis* ST376 harbouring OptrA RDK variant (isolate ID AT34), has also been detected in human clinical and environmental isolates in China, and in fattening pigs in Switzerland [13,24,32]. *E. faecalis* ST593 carrying OptrA EYDNDM (isolates AT09, AT29, AT46a, and AT49a, respectively) has also been identified in clinical isolates in China [25]. Moreover, *E. faecalis* ST1008 co-harbouring *optrA* and *poxtA* has been identified in several raw pet food samples that are commercially available across different countries in Europe [18]. Lastly, *E. faecium* ST168 carrying *poxtA* has previously been found in two human clinical isolates in Caen, France [33].

Notably, while many previously described *E. faecalis* and *E. faecium* with ST or oxazolidinone resistance genes identical to those we identified exhibit phenotypical resistance to linezolid [8,9,33-35], the only phenotypically resistant isolates according to CLSI definitions in the current investigation were *E. faecalis* ST256 (isolate AT50) harbouring OptrA KD, and *V. lutrae* (isolate AT15) harbouring OptrA EDM [14]. This finding is supportive of previous studies that observe divergent linezolid MIC values among enterococcal species, their STs, and the OptrA variants or *poxtA* genes they harbour [13,34]. While it has been suggested that bacterial host factors may play a role in the expression of linezolid resistance genes [34], there is currently insufficient understanding of the mechanisms that determine the resistance phenotype of Gram-positive bacterial species harbouring linezolid resistance determinants. Although both linezolid resistant isolates in this study harboured *optrA* on plasmids, no obvious correlation of resistance phenotype with genomic location of the resistance determinants can be made using the data from this study, and further investigations would be required to determine the impact of gene locations on the observed resistance phenotypes of the host bacteria. The lack of a linezolid resistance phenotype among many isolates in this study indicates that our selective approach using florfenicol to detect linezolid resistance mechanisms in enterococci and other Gram-positive bacterial species may be helpful for screening meat and meat-based products.

Finally, it is important to highlight some of the potential limitations of our study. First, results should be interpreted considering our sampling strategy, which was convenience-based. This led to only representation of RMBD suppliers from Germany and Switzerland, and meats originating mostly from these two countries. Thus, the results cannot be generalised to RMBD products that are available from other suppliers, or RMBDs produced using meat from other countries in Europe. Second, sample sizes were small and there were unequal sample sizes among the suppliers and among the different meat types. Therefore, there remains the possibility of having introduced unintended selection bias.

## Conclusions

The occurrence of isolates harbouring linezolid resistance genes in raw dog food highlights the importance of promoting awareness of the possible risks associated with RMBDs and of providing information to pet owners on correct handling and feeding of RMBDs in order to mitigate potential health risks**.**

## References

[1] Davies RH Lawes JR Wales AD . Raw diets for dogs and cats: a review, with particular reference to microbiological hazards. J Small Anim Pract. 2019;60(6):329-39. 10.1111/jsap.13000 31025713 PMC6849757

[2] Morelli G Bastianello S Catellani P Ricci R . Raw meat-based diets for dogs: survey of owners’ motivations, attitudes and practices. BMC Vet Res. 2019;15(1):74. 10.1186/s12917-019-1824-x 30832667 PMC6399943

[3] Fredriksson-Ahomaa M Heikkilä T Pernu N Kovanen S Hielm-Björkman A Kivistö R . Raw meat-based diets in dogs and cats. Vet Sci. 2017;4(3):33. 10.3390/vetsci4030033 29056692 PMC5644655

[4] World Health Organization (WHO). Global antimicrobial resistance and use surveillance system (GLASS) report: 2021. Geneva: WHO; 2021. Available from: https://www.who.int/publications/i/item/9789240027336

[5] Aarestrup FM . The livestock reservoir for antimicrobial resistance: a personal view on changing patterns of risks, effects of interventions and the way forward. Philos Trans R Soc Lond B Biol Sci. 2015;370(1670):20140085. 10.1098/rstb.2014.0085 25918442 PMC4424434

[6] Kehrenberg C Schwarz S . fexA, a novel Staphylococcus lentus gene encoding resistance to florfenicol and chloramphenicol. Antimicrob Agents Chemother. 2004;48(2):615-8. 10.1128/AAC.48.2.615-618.2004 14742219 PMC321516

[7] Antonelli A D’Andrea MM Brenciani A Galeotti CL Morroni G Pollini S Characterization of poxtA, a novel phenicol-oxazolidinone-tetracycline resistance gene from an MRSA of clinical origin. J Antimicrob Chemother. 2018;73(7):1763-9. 10.1093/jac/dky088 29635422

[8] Wang Y Lv Y Cai J Schwarz S Cui L Hu Z A novel gene, optrA, that confers transferable resistance to oxazolidinones and phenicols and its presence in Enterococcus faecalis and Enterococcus faecium of human and animal origin. J Antimicrob Chemother. 2015;70(8):2182-90. 10.1093/jac/dkv116 25977397

[9] Schwarz S Zhang W Du XD Krüger H Feßler AT Ma S Mobile oxazolidinone resistance genes in Gram-positive and Gram-negative bacteria. Clin Microbiol Rev. 2021;34(3):e0018820. 10.1128/CMR.00188-20 34076490 PMC8262807

[10] World Health Organization (WHO). Critically important antimicrobials for human medicine. 6th revision: 2019. Geneva: WHO; 2019. Available from: https://www.who.int/publications/i/item/9789241515528

[11] Timmermans M Bogaerts B Vanneste K De Keersmaecker SCJ Roosens NHC Kowalewicz C Large diversity of linezolid-resistant isolates discovered in food-producing animals through linezolid selective monitoring in Belgium in 2019. J Antimicrob Chemother. 2021;77(1):49-57. 10.1093/jac/dkab376 34673924 PMC8730767

[12] Freitas AR Tedim AP Duarte B Elghaieb H Abbassi MS Hassen A Linezolid-resistant (Tn6246:fexB-poxtA) Enterococcus faecium strains colonizing humans and bovines on different continents: similarity without epidemiological link. J Antimicrob Chemother. 2020;75(9):2416-23. 10.1093/jac/dkaa227 32607549

[13] Nüesch-Inderbinen M Haussmann A Treier A Zurfluh K Biggel M Stephan R . Fattening pigs are a reservoir of florfenicol-resistant enterococci harboring oxazolidinone resistance genes. J Food Prot. 2022;85(5):740-6. 10.4315/JFP-21-431 35258564

[14] Clinical and Laboratory Standards (CLSI). Performance standards for antimicrobial susceptibility testing. Thirty-second edition. Wayne, PA; CLSI supplement M100. 2022.

[15] Kehrenberg C Schwarz S . Distribution of florfenicol resistance genes fexA and cfr among chloramphenicol-resistant Staphylococcus isolates. Antimicrob Agents Chemother. 2006;50(4):1156-63. 10.1128/AAC.50.4.1156-1163.2006 16569824 PMC1426988

[16] Guerin F Sassi M Dejoies L Zouari A Schutz S Potrel S Molecular and functional analysis of the novel cfr(D) linezolid resistance gene identified in Enterococcus faecium. J Antimicrob Chemother. 2020;75(7):1699-703. 10.1093/jac/dkaa125 32277823

[17] Zhu Y Yang W Schwarz S Xu Q Yang Q Wang L Characterization of the novel optrA-carrying pseudo-compound transposon Tn7363 and an Inc18 plasmid carrying cfr(D) in Vagococcus lutrae. J Antimicrob Chemother. 2022;77(4):921-5. 10.1093/jac/dkab478 35038329

[18] Freitas AR Finisterra L Tedim AP Duarte B Novais C Peixe L from the ESCMID Study Group on Food- and Water-borne Infections (EFWISG) . Linezolid- and multidrug-resistant enterococci in raw commercial dog food, Europe, 2019-2020. Emerg Infect Dis. 2021;27(8):2221-4. 10.3201/eid2708.204933 34287135 PMC8314808

[19] Chen M Pan H Lou Y Wu Z Zhang J Huang Y Epidemiological characteristics and genetic structure of linezolid-resistant *Enterococcus faecalis.* Infect Drug Resist. 2018;11:2397-409. 10.2147/IDR.S181339 30538507 PMC6251436

[20] Tsilipounidaki K Gerontopoulos A Papagiannitsis C Petinaki E . First detection of an *optrA*-positive, linezolid-resistant ST16 *Enterococcus faecalis* from human in Greece. New Microbes New Infect. 2019;29:100515. 10.1016/j.nmni.2019.01.010 30899521 PMC6406052

[21] Vorobieva V Roer L Justesen US Hansen F Frimodt-Møller N Hasman H Detection of the optrA gene in a clinical ST16 Enterococcus faecalis isolate in Denmark. J Glob Antimicrob Resist. 2017;10:12-3. 10.1016/j.jgar.2017.05.002 28572038

[22] Zhou W Gao S Xu H Zhang Z Chen F Shen H Distribution of the optrA gene in Enterococcus isolates at a tertiary care hospital in China. J Glob Antimicrob Resist. 2019;17:180-6. 10.1016/j.jgar.2019.01.001 30641287

[23] Kerschner H Rosel AC Hartl R Hyden P Stoeger A Ruppitsch W Oxazolidinone resistance mediated by *optrA* in clinical *Enterococcus faecalis* isolates in Upper Austria: first report and characterization by whole genome sequencing. Microb Drug Resist. 2021;27(5):685-90. 10.1089/mdr.2020.0098 33090061

[24] Cui L Wang Y Lv Y Wang S Song Y Li Y Nationwide surveillance of novel oxazolidinone resistance gene *optrA* in *Enterococcus* isolates in China from 2004 to 2014. Antimicrob Agents Chemother. 2016;60(12):7490-3. 10.1128/AAC.01256-16 27645239 PMC5119033

[25] Cai J Wang Y Schwarz S Lv H Li Y Liao K Enterococcal isolates carrying the novel oxazolidinone resistance gene optrA from hospitals in Zhejiang, Guangdong, and Henan, China, 2010-2014. Clin Microbiol Infect. 2015;21(12):1095.e1-4. 10.1016/j.cmi.2015.08.007 26319902

[26] Jung YH Cha MH Woo GJ Chi YM . Characterization of oxazolidinone and phenicol resistance genes in non-clinical enterococcal isolates from Korea. J Glob Antimicrob Resist. 2021;24:363-9. 10.1016/j.jgar.2021.01.009 33515778

[27] Kim E Shin S-W Kwak H-S Cha M-H Yang S-M Gwak Y-S Prevalence and characteristics of phenicol-oxazolidinone resistance genes in *Enterococcus faecalis* and *Enterococcus faecium* isolated from food-producing animals and meat in Korea. Int J Mol Sci. 2021;22(21):11335. 10.3390/ijms222111335 34768762 PMC8583520

[28] Li J Yang L Huang X Wen Y Zhao Q Huang X Molecular characterization of antimicrobial resistance and virulence factors of Enterococcus faecalis from ducks at slaughterhouses. Poult Sci. 2022;101(4):101646. 10.1016/j.psj.2021.101646 35172230 PMC8851247

[29] Gião J Leão C Albuquerque T Clemente L Amaro A . Antimicrobial susceptibility of Enterococcus isolates from cattle and pigs in Portugal: Linezolid resistance genes optrA and poxtA. Antibiotics (Basel). 2022;11(5):615. 10.3390/antibiotics11050615 35625259 PMC9137492

[30] Nüesch-Inderbinen M Raschle S Stevens MJA Schmitt K Stephan R . Linezolid-resistant Enterococcus faecalis ST16 harbouring optrA on a Tn6674-like element isolated from surface water. J Glob Antimicrob Resist. 2021;25:89-92. 10.1016/j.jgar.2021.02.029 33705941

[31] McHugh MP Parcell BJ Pettigrew KA Toner G Khatamzas E El Sakka N Presence of *optrA-*mediated linezolid resistance in multiple lineages and plasmids of *Enterococcus faecalis* revealed by long read sequencing. Microbiology (Reading). 2022;168(2):168. 10.1099/mic.0.001137 35130141 PMC8941993

[32] Yu Y Ye XQ Liang HQ Zhong ZX Cheng K Sun J Lilium spp., as unnoticed environmental vector, spreading OptrA-carrying Enterococcus spp. Sci Total Environ. 2022;816:151540. 10.1016/j.scitotenv.2021.151540 34767892

[33] Dejoies L Sassi M Schutz S Moreaux J Zouari A Potrel S Genetic features of the poxtA linezolid resistance gene in human enterococci from France. J Antimicrob Chemother. 2021;76(8):1978-85. 10.1093/jac/dkab116 33895846

[34] Cai J Schwarz S Chi D Wang Z Zhang R Wang Y . Faecal carriage of optrA-positive enterococci in asymptomatic healthy humans in Hangzhou, China. Clin Microbiol Infect. 2019;25(5):630.e1-6. 10.1016/j.cmi.2018.07.025 30076974

[35] Freitas AR Tedim AP Novais C Lanza VF Peixe L . Comparative genomics of global *optrA*-carrying *Enterococcus faecalis* uncovers a common chromosomal hotspot for *optrA* acquisition within a diversity of core and accessory genomes. Microb Genom. 2020;6(6):6. 10.1099/mgen.0.000350 32149599 PMC7371108

